# Hypogonadism in Patients with Prader Willi Syndrome: A Narrative Review

**DOI:** 10.3390/ijms22041993

**Published:** 2021-02-17

**Authors:** Luigi Napolitano, Biagio Barone, Simone Morra, Giuseppe Celentano, Roberto La Rocca, Marco Capece, Vincenzo Morgera, Carmine Turco, Vincenzo Francesco Caputo, Gianluca Spena, Lorenzo Romano, Luigi De Luca, Gianluigi Califano, Claudia Collà Ruvolo, Francesco Mangiapia, Vincenzo Mirone, Nicola Longo, Massimiliano Creta

**Affiliations:** Department of Neurosciences, Reproductive Sciences and Odontostomatology, University of Naples “Federico II”, 80131 Naples, Italy; luigi.napolitano12@studenti.unina.it (L.N.); biagio.barone@unina.it (B.B.); simone.morra@unina.it (S.M.); Roberto.larocca@unina.it (R.L.R.); marco.capece@unina.it (M.C.); vincenzo.morgera@unina.it (V.M.); carmine.turco2@unina.it (C.T.); vincenzofrancesco.caputo@unina.it (V.F.C.); gia.spena@studenti.unina.it (G.S.); Lorenzo.romano@unina.it (L.R.); luigi.deluca@unina.it (L.D.L.); Gianluigi.califano@unina.it (G.C.); claudia.collaruvolo@unina.it (C.C.R.); Francesco.mangiapia@unina.it (F.M.); mirone@unina.it (V.M.); nicola.longo@unina.it (N.L.); Massimiliano.creta@unina.it (M.C.)

**Keywords:** Prader-Willi syndrome, hypogonadism, chromosome 15 abnormalities, genomic imprinting

## Abstract

Prader-Willi syndrome (PWS) is a multisystemic complex genetic disorder related to the lack of a functional paternal copy of chromosome 15q11-q13. Several clinical manifestations are reported, such as short stature, cognitive and behavioral disability, temperature instability, hypotonia, hypersomnia, hyperphagia, and multiple endocrine abnormalities, including growth hormone deficiency and hypogonadism. The hypogonadism in PWS is due to central and peripheral mechanisms involving the hypothalamus-pituitary-gonadal axis. The early diagnosis and management of hypogonadism in PWS are both important for physicians in order to reach a better quality of life for these patients. The aim of this study is to summarize and investigate causes and possible therapies for hypogonadism in PWS. Additional studies are further needed to clarify the role of different genes related to hypogonadism and to establish a common and evidence-based therapy.

## 1. Epidemiology

Prader-Willi Syndrome (PWS) is a rare genetic disorder described for the first time by Prader, Labhart, and Willi in 1956 [1]. It has an estimated prevalence in the world population of 1/10,000–1/30,000 with over 400,000 patients currently affected worldwide [2,3]. It occurs equally in males and females of all races [4]. Although most PWS cases are sporadic, some familial cases have been described, with a recurrence risk up to 50% [5]. PWS carries a risk of significant mortality in both children and adults. Whittington et al. reported a mortality rate for the PWS population of 3% per year across the age range of 0 to 47 years old and of 7% per year for patients aged >30 years [6]. The most common causes of death in children are respiratory infections and sudden death [7]. Mortality in adults has been related to obesity and its complications, including cardiovascular diseases, sleep apnea, diabetes mellitus, and hypertension [8]. Despite being particularly recognizable, the PWS phenotype evolves throughout life, thus requiring ongoing clinical evaluation of medical issues [5]. Hypogonadism is one of the most important clinical features of PWS [9].

## 2. Molecular Genetics

PWS is caused by a lack of expression of genes classified as imprinted in the region of chromosome 15q11.2-q131 (PWS region) [10]. The 15q11.2-q13 region is vulnerable to several rearrangements, such as deletion, translocation, duplications, and epigenetic changes causing PWS. In 1981, Ledbetter et al. described, for the first time, an interstitial deletion in the proximal long arm of chromosome 15 as the genetic basis for PWS [11]. Subsequently, several genes have been described in the PWS region. Most of these genes are subject to genomic imprinting. Thus, only the alleles from the paternally derived chromosome are expressed, while the same alleles from the maternally derived chromosome 15 are silenced [12].

### 2.1. Chromosomal Abnormalities

**Figure 1:**
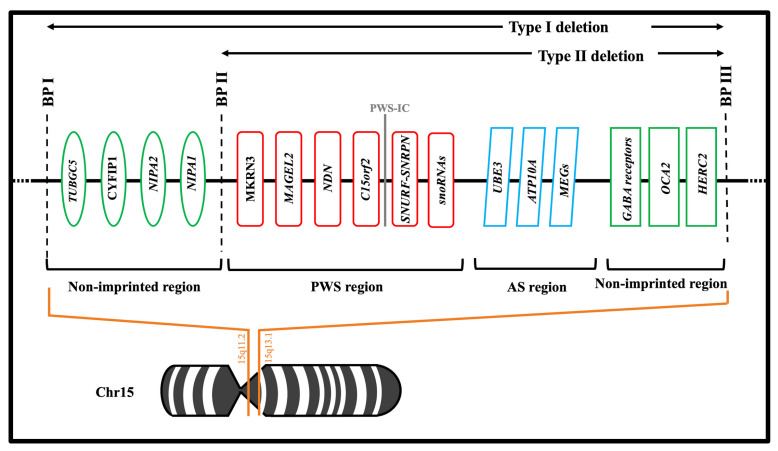
The Ideogram of Chromosome 15q11-q13 showing main genes in PWS located in the typical deletion region of Prader-Willi syndrome. BP, breakpoint; PWS, Prader-Willi syndrome; PWS-IC, Prader-Willi syndrome-imprinting center; AS, Angelman Syndrome. Green Ovals and Rectangles: Biallelic expression; Red Rounded rectangles: Paternal expression; Light Blue Parallelograms: Maternal expression. Adapted from Cheon et al. [16].

There are three main molecular mechanisms involving the PWS region and responsible for the PWS syndrome: the most common is chromosome deletion (up to 65–75%), followed by maternal uniparental disomy (20–30%), imprinting center defects (1–3%) and rare chromosomal translocations (2%) [13,14,15]. The 15q11.2-q13 region can be divided into four segments delineated by three common deletion breakpoints [14]. The first region contains *TUBGCP5*, *CYPFIP1*, *NIPA2* and *NIPA1* genes, and it is proximal to a non-imprinted region between breakpoints one and two (BPI, BPII) [16]. These genes are equally expressed from paternal and maternal alleles. The second region contains the PWS domain, with only paternally expressed protein-coding genes *MKRN3*, *MAGEL2*, *NDN*, *C15orf2*, *SNRPN-SNURF* and *snoRNAs* [17]. The third region is the Angelman Syndrome domain, which contains preferentially maternally expressed genes (*UBE3*, *ATP10A* and *MEGs*) [16]. The fourth and last region is a distal non-imprinted region containing the gene for oculocutaneous albinism type 2 (*OCA2*), the gene *HERC2*, and three gamma-aminobutyric acid (GABA) receptor genes and the distal breakpoint (BPIII) [16,18,19] (Figure 1). According to the literature, two types of deletion have been reported: type I and type II. Type I occurs in approximately 40% of cases between BP I and BP III whereas Type II occurs in 60% of cases between BP II and BP III [18]. Both types of deletions are almost universal de novo events with the Type I subgroup showing a more severe phenotype than Type II [20].

Maternal uniparental disomy has been described in approximately 20–30% of patients, although this percentage increases with maternal age [19]. Three disomy types have been described: heterodisomy 15 (two different chromosome 15s from the mother due to errors in meiosis I); isodisomy 15 (two identical chromosome 15s due to errors in meiosis II); isodisomy 15 with two partially different chromosome 15s from the mother due to errors in meiosis I from nondisjunction and crossover events leading to segments of isodisomy or loss of heterozygosity. An imprinting center defect occurs in about 5% and reportedly causes abnormal imprinting or methylation that silences paternal genes in the PWS region [13,14,15].

### 2.2. Encoded Genes

Several imprinted genes expressed exclusively from the paternal chromosome are believed to be involved in this syndrome. The genes commonly investigated include: *SNURF-SNRPN*, *NDN*, *MAGEL2*, *MKRN3*, *C15orf2* and *HBII snoRNA* cluster [21]. Results from animal models demonstrate that several of these genes are involved in neural development, brain function, infertility, and circadian rhythm. In detail, *MKRN3*, *MAGEL2* and *NDN* are three genes located proximally to the imprinting center (IC) in the *SNRPN-SNURF* gene complex and are involved in neural development and brain function [22]. *SNURF-SNRPN*, a bicistronic gene, forms a complex gene locus with 148 possible exons that undergo alternative splicing and encode two different proteins: exon 1–3 encodes for *SNURF* and produces a polypeptide of unknown function; exon 4–10 encodes for SmN, a spliceosome protein involved in mRNA splicing [23]. This gene, in particular, serves as a host for six snoRNA genes which are regulated by the expression of *SNURF-SNRPN* and do not encode proteins [24]. *MAGEL2* encodes a protein located in the hypothalamus and other cerebral areas. Its involvement has been found recently in autism spectrum disorders, brain structure development, human reproduction, and infertility [25]. In addition, *MAGEL2* is considered a gene linked to eating disorders such as hyperphagia and its mutations are connected to PWS phenotype [26,27]. *MKRN3* encodes proteins called makorin ring finger protein 3, expressed in the brain. The physiological and functional role of this gene still needs to be fully understood given its collocation in the PWS region. However, it seems to be important for hormone regulation and familial central precocious puberty (CPP) [28,29]. *C15orf2* encodes an 1156-amino-acid protein of unknown function, present in primates only and biallelically expressed in brain and adult testis [30]. Although its role is still unclear, it is suggested that *C15orf2* regulates several spermatid-specific genes and, due to its position in chromosome 15 and its susceptibility to genomic imprinting, it is speculated that defects of this gene could impair spermatogenesis and male fertility in PWS patients [31]. *NDN*, located in a large, imprinted domain and with its maternally inherited allele normally silenced, encodes the *MAGE* family protein Necdin. *NDN* is highly expressed in mature hypothalamic neurons (in particular GnRH neuronal cells) and seems to contribute to the hypogonadotropic hypogonadal phenotype in PWS [32,33]. The murine model lacking Necdin have, indeed, reduced numbers of gonadotropin-releasing hormone (GnRH) neurons [34]. Further data suggest that Necdin activates GnRH transcription and is necessary for full GnRH neuronal development [35]. Another gene *SNORD 116* is expressed in the hypothalamic appetite control center. This deletion causes pro convertase 1 (PC1) deficiency. PC1 is a protein involved in several hormonal pathways and this may explain the hormonal changes found in PWS: Growth hormone-releasing hormone (GHRH) (growth hormone deficiency and short stature); proGnRH (hypogonadism); progrelin (hypergrelinemia); proinsulin (relative hypoinsulinemia and type 2 diabetes mellitus); proopiomelanocortin—POMC (hypocortisolism); and ProTRH (hypothyroidism) [36]. Currently, however, their function and expression patterns in humans are not well characterized [13,14,15,16,17,18,37,38,39] (Table 1).

## 3. Hypogonadism in PWS

### 3.1. Pathophysiology

The pathophysiology of hypogonadism in PWS patients is complex and still represents a matter of debate. Traditionally, hypogonadism has been considered a result of hypothalamic dysfunction (congenital hypogonadotropic hypogonadism) due to neuronal dysfunction involving LH-releasing hormone (LHRH) neurons with subsequent impairment of gonadotropins and sex steroid secretion [2]. On the contrary, other studies have suggested that a primary gonadal failure, rather than a hypothalamic dysfunction, could contribute to hypogonadism in PWS patients, with subsequent abnormal pubertal development and infertility [2,3,40,41,42,43]. 

Siemensma et al. reported normal inhibin B levels in PWS male patients up to 10 years of age while between 10–15 years patients start to show high levels of FSH (Follicle-stimulating hormone) with decreased Inhibin B and testosterone levels [40]. These findings led to suspect a post puberal primary failure of Sertoli cells (which are responsible for inhibin B production) confirmed in histologic reports, despite no correlation between testicular histology and severity of hypogonadism [2,44]. Spermatogonia are indeed present in prepuberal patients with favorable testicular histology, nevertheless potential fertility could be impaired by subsequent testicular dysfunction, as testified by persisting increased FSH levels [42]. 

Female patients with PWS showed analogous patterns with low levels of inhibin B and anti-Mullerian hormone (AMH) (both produced by the granulosa cells in follicles during primary and preantral stages) with normal FSH levels and normal size of primordial and antral follicle pool; only a small percentage of patients showed normal or slightly decreased inhibin B and AMH levels with the development of a dominant follicle [45,46,47]. Antagonization of estrogens in those patients induced a considerable secretion of FSH and LH, excluding dysfunctional hypothalamic-pituitary-gonadal feedback, thus suggesting, as in males, a variable combination of primary ovarian and hypothalamic defects [48]. As results of this variability of combinations of gonadal and central dysfunction, Gross-Tsur et al. defined four different distinct phenotypes of hypogonadism based on the analysis of FSH and inhibin B levels, ranging from the most common primary gonadal dysfunction to the rarer central hypogonadism [49].

### 3.2. Clinical Presentation of Hypogonadism in PWS

From a clinical point of view, hypogonadism in PWS is characterized by genital hypoplasia in both sexes, cryptorchidism in males, pubertal insufficiency, and infertility [2,47,50]. Genital abnormalities are the rule in PWS patients. These features are typically evident in males and could be less evident in females. Table 2 itemizes the manifestations of hypogonadism in males and females across development. 

Cryptorchidism, in particular, is present in 85–100% of cases and almost always requires surgical orchidopexy, which permits 40% of patients to reach normal/subnormal testes volume >7 mL [51]. In most male patients, mini puberty occurs normally with a transient increase in gonadotropins and testosterone levels during the first months of life [52,53]. Pubertal development rarely progresses over Tanner genital stages 2–3, resulting in low testicular volume and delayed or absent sexual maturation with absence of facial/body hair growing and voice change [48]. However, rare cases of premature puberty during growth hormone replacement therapy are reported in the literature; in such eventualities, the use of a GnRH analogs (usually 3.75 mg or 11.5 mg intramuscular leuprorelin) enabled the attainment of a reasonable adult stature, restoring a more appropriate pubertal development [54,55]. The penile length shows some improvement during adolescence with normal values until 10–12 years. However, it remains below normal ranges thereafter. Other abnormalities include scrotal hypoplasia with hypopigmented, thin, and poorly rugate skin [56]. 

Clinical manifestations of hypogonadism in females are more heterogeneous with hypoplasia or absence of labia minora and/or clitoris representing the most common features encountered in about 70% of cases [12]. Similar to the male counterpart, mini puberty is normal in infant girls and pubertal development starts spontaneously in over 80% of cases at normal age, with anecdotal cases of precocious puberty [57,58]. Moreover, spontaneous menarche occurs in 25–44% of patients [48,56,59]. However, the majority of patients subsequently develop amenorrhea or oligomenorrhea with occasional vaginal bleeding [60,61]. With gonadotropin levels generally in normal ranges, breasts could develop up to Tanner 5 stage, although many patients have low estradiol and inhibin B levels [55]. Particularly relevant is that fertility could be feasible with inhibin B levels >20 pg/mL [62], thus potential contraception methods should be suggested in those cases [63]. 

Premature adrenarche presents a high prevalence in PWS patients of both sexes, with up to 30% of cases involved and it is commonly associated with a non-progressive pubic or axillary hair growth [59]. Despite the fact that obesity and impaired insulin sensitivity is thought to trigger premature adrenarche, no difference was observed with non-syndromic obese patients and in those treated with rhGH (recombinant human growth hormone), thus questioning the influence of the metabolic profile on premature adrenal activity in PWS patients [64].

### 3.3. Laboratory Data and Hormones Levels

LH and FSH in PWS males start to increase at 8–10 years up to reach normal levels in adolescence with few exceptions that show increased FHS levels. Relatively few variations are seen in adult men for LH levels, whereas FSH levels start to increase after 20 years, remaining persistently above the normal range. Testosterone levels fall from normal mini puberty values to the low or undetectable range during childhood, and despite an increase in adolescence, testosterone levels remain below normal range. Similarly, Inhibin B and SHBG (sex hormone binding globulin) remain below normal ranges after childhood [48,53].

In females, LH and FSH are relatively high during infancy, starting to drop to low levels in early childhood and re-increase in adolescence and adulthood up to normal ranges. However, many exceptions are reported with a variable combination of levels for these gonadotropins. Estradiol levels follow a normal pattern with high levels in infancy, lower values in childhood and adolescence, and normal/low levels in adulthood [48,63].

Conventional GnRH stimulation consists of intravenous administration of LH-RH of dose 100 mcg/m^2^ or 2.5 mcg/kg and determination of peak LH and FSH responses in five to eight blood samples in approximately 20–30 min, which makes it cumbersome and costly. [65]

### 3.4. Therapy for Hypogonadism in PWS

Due to the improved recognition of the condition and the increasing availability of testing, PWS is being diagnosed earlier, allowing early access to treatments [66,67]. The mainstay of therapy for hypogonadism in PWS patients is substitutive hormonal therapy. The aim of substitutive therapies in PWS is indeed to achieve a satisfactory development of patients (both in terms of mental and physical development) and a recognizable improved quality of life. The following strategies can be adopted to manage PWS patients: gonadotropin or sex hormones [62]. The first hypogonadism therapy proposed in male patients with PWS was the use of human chorionic gonadotropin (hCG), intending to stimulate spontaneous testicular descent and to increase the scrotal size and penile length. Although results regarding spontaneous testicular descent were poor, 250–500 IU twice weekly of hCG confirmed its efficacy in scrotal and penile development, showing a sustained increase of testosterone levels, which persisted below normal ranges but permitted the development of an adult pubic hair pattern [68]. Similarly, Eiholzer et al. reported in eight patients treated with hCG (500–1500 IU twice weekly) an increased testosterone level (up to 2.8-fold compared to base level) and decreased FSH level, inducing clinical signs of androgenization (deepening of the voice and genital development) during the follow up (mean 3 years) [69]. The testicular volume also increased slightly (up to 6 mL) but remained below normal ranges while no effects were reported on inhibin B levels. 

Currently, no shared guidelines nor strong evidence are reported for hypogonadism treatments in PWS patients, thus leaving the treatment of this particular condition in an experimental stage. Nowadays, the use of gonadotropin or sex hormones are considered potential strategies useful to improve hypogonadism and sexual function [70,71]. The use of sex steroids as hypogonadism replacement therapy is suggested by several studies, promoting improvements in bone density and muscle mass and inducing overall well-being via effects on body image, quality of life, and social relations [63,72]. Sex hormone replacement should be considered in patients with PWS where hypogonadism is more severe. 

Testosterone replacement therapy represents the standard of care in male patients with PWS. Lacking a standardized regimen, treatment is extrapolated from other forms of hypogonadism. In everyday clinical practice, injectable testosterone formulations are preferred due to their convenience and cost [19,52]. Testosterone patches and gel preparations offer the advantage of avoiding the peaks and troughs of injections. However, these alternatives are more expensive and require daily administration, raising problems of adherence and the risk for skin irritation [19,53]. Experts agree that the timing and dosing of testosterone replacement should reflect the normal process of puberty. However, no consensus exists about the most appropriate regimen for pubertal induction and for hormone replacement in adults. Heksch et al. recommend treatment with intramuscular testosterone in males with delayed or incomplete puberty, usually by age 15–16 years and starting at a dose of 50–100 mg given every 28 days with gradual increase towards typical adult male doses [62]. Once males are at adult doses, testosterone patches or gel could be considered, although caution must be taken in individuals with skin picking tendencies [62]. It has been reported that 125 mg of monthly intramuscular testosterone, the half dose used for other forms of hypogonadism, increased body hair in 77.3% patients (17 on 22), induced erectile function in 36.4% (8/22) and ejaculation in 13.6% (3/22) despite no sperm being found in semen; no adverse effects or increased aggressivity was reported for the regimen used [73]. Adequate replacement therapy should be started in adolescence, however, considering this a critical period in PWS and the presence of psychotic episodes in one fifth of young adults during replacement hormonal therapy, particular caution should be provided when administering testosterone replacement therapy even in patients without self-injury tendencies, due to the possibility of exacerbating or precipitating aggression in adolescent PWS patients [15,68].

Substitutive therapy in females has to be more tailored, due to a broader spectrum of symptoms which are bound to the residual hormonal activity, which ranges from severe gonadal dysfunction to fully developed with potential fertility. Proper assessment of the hormonal status of patients should be obtained before any therapeutic choice is determined, with particular attention to inhibin B levels. Estrogen therapy consisting of low levels of estradiol has been recommended in females with amenorrhea/oligomenorrhea [56]. Cyclic progesterone treatment is suggested for women with irregular vaginal bleeding/oligomenorrhea and normal estradiol levels, while combined estrogen/progesterone replacement treatment is suggested for women with amenorrhea and low estradiol levels [63].

## 4. Conclusions

PWS is a complex disease with hypogonadism a common finding in PWS patients. The pathophysiology of hypogonadism is probably multifactorial and still under debate. The mainstay of therapy for hypogonadism in PWS patients is substitutive hormonal therapy involving testosterone replacement therapy in males and estrogen therapy in females. Although the advantages of hormonal replacement therapy are evident, such as improved quality of life, increased bone density, and muscle mass together with a proper development of secondary sexual characteristics, some concerns persist, such as increased aggressivity in males and potential lack of usefulness in females with conserved hormonal activity. Further studies are necessary to better clarify the mechanisms of hypogonadism, the role of the different genes involved in this syndrome, and in particular, to delineate an appropriate, standardized approach to therapy.

## Figures and Tables

**Table 1:** Genes, their function, and their potential role in PWS.

Gene	Function	Potential Role in PWS
*MKRN3*	Hormonal regulation, familial central precocious puberty (CPP) [28,29]	↓ Hypothalamic GnRH secretion [29]
*MAGEL2*	Brain structure development, human reproduction, fertility [25]	Eating disorder (hyperphagia) [26,27]
*NDN*	GnRH neurons development [34]	Hypogonadotropic hypogonadal phenotype [2,32]
*SNORD116*	ProConvertase 1 (PC) activation and regulation of some hormonal pathways [36]	hormonal changes: -↓GHRH → short stature [36] -↓ProGnRH → Hypogonadism [36] -↓ProGrelin → Hypergrelinemia [36] -↓Proinsulin → Hypoinsulinemia and DM2 [36] -↓ Proopiomelanocortin → Hypocortisolism [36] -↓ProTRH → Hypothyroidism [36]
*C15orf2*	Regulation of several spermatid-specific genes [31]	Impaired spermatogenesis and male fertility [31]

GnRH: Gonadotropin-releasing hormone, TRH: thyrotropin releasing hormone, ↓: reduction.

**Table 2:** Manifestations of hypogonadism.

Male	Female
Scrotal Hypoplasia [56]	Hypoplasia of Labia Minora and/or clitoris [48]
Cryptorchidism [51]	Delayed spontaneous puberal development with menarche [48,59]
Low penile length [59]	Amenorrhea or oligomenorrhea [60,61]
Low serum levels of Testosterone and Inhibin B [59]	Low serum levels of Estradiol and Inhibin B [53]
Tanner stage 3–4 [48]	Tanner stage 3–4 [62]

## Data Availability

Not applicable.
